# Gelatin/Nanohyroxyapatite Cryogel Embedded Poly(lactic-*co*-glycolic Acid)/Nanohydroxyapatite Microsphere Hybrid Scaffolds for Simultaneous Bone Regeneration and Load-Bearing

**DOI:** 10.3390/polym10060620

**Published:** 2018-06-05

**Authors:** K. T. Shalumon, Chang-Yi Kuo, Chak-Bor Wong, Yen-Miao Chien, Huai-An Chen, Jyh-Ping Chen

**Affiliations:** 1 Department of Chemical and Materials Engineering, Chang Gung University, Kwei-San, Taoyuan 33302, Taiwan; shalumon@gmail.com (K.T.S.); onesky1997@gmail.com (C.-Y.K.); dabubuya@gmail.com (Y.-M.C.); eam012331@gmail.com (H.-A.C.); 2 Department of Orthopaedic Surgery, Chang Gung Memorial Hospital, Keelung 20401, Taiwan; ivanbor@adm.cgmh.org.tw; 3 Department of Plastic and Reconstructive Surgery and Craniofacial Research Center, Chang Gung Memorial Hospital, Kwei-San, Taoyuan 33305, Taiwan; 4 Research Center for Food and Cosmetic Safety, Research Center for Chinese Herbal Medicine, Chang Gung University of Science and Technology, Kwei-San, Taoyuan 33302, Taiwan; 5 Department of Materials Engineering, Ming Chi University of Technology, Tai-Shan, New Taipei City 24301, Taiwan

**Keywords:** poly(lactic-*co*-glycolic acid), nanohydroxyapatite, cryogel, microsphere, hybrid scaffold, bone tissue engineering, gelatin

## Abstract

It is desirable to combine load-bearing and bone regeneration capabilities in a single bone tissue engineering scaffold. For this purpose, we developed a high strength hybrid scaffold using a sintered poly(lactic-*co*-glycolic acid) (PLGA)/nanohydroxyapatite (nHAP) microsphere cavity fitted with gelatin/nHAP cryogel disks in the center. Osteo-conductive/osteo-inductive nHAP was incorporated in 250–500 μm PLGA microspheres at 40% (*w*/*w*) as the base matrix for the high strength cavity-shaped microsphere scaffold, while 20% (*w*/*w*) nHAP was incorporated into gelatin cryogels as an embedded core for bone regeneration purposes. The physico-chemical properties of the microsphere, cryogel, and hybrid scaffolds were characterized in detail. The ultimate stress and Young’s modulus of the hybrid scaffold showed 25- and 21-fold increases from the cryogel scaffold. In vitro studies using rabbit bone marrow-derived stem cells (rBMSCs) in cryogel and hybrid scaffolds through DNA content, alkaline phosphatase activity, and mineral deposition by SEM/EDS, showed the prominence of both scaffolds in cell proliferation and osteogenic differentiation of rBMSCs in a normal medium. Calcium contents analysis, immunofluorescent staining of collagen I (COL I), and osteocalcin (OCN) and relative mRNA expression of COL I, OCN and osteopontin (OPN) confirmed in vitro differentiation of rBMSCs in the hybrid scaffold toward the bone lineage. From compression testing, the cell/hybrid scaffold construct showed a 1.93 times increase of Young’s modulus from day 14 to day 28, due to mineral deposition. The relative mRNA expression of osteogenic marker genes COL I, OCN, and OPN showed 5.5, 18.7, and 7.2 folds increase from day 14 to day 28, respectively, confirming bone regeneration. From animal studies, the rBMSCs-seeded hybrid constructs could repair mid-diaphyseal tibia defects in rabbits, as evaluated by micro-computed tomography (μ-CT) and histological analyses. The hybrid scaffold will be useful for bone regeneration in load-bearing areas.

## 1. Introduction

According to location and function, the mechanical properties of bone varies from one area to another in the human body [1]. Considering the complex and classified structure, bone could be considered as a heterogeneous composite consisting of a mineral phase (hydroxyapatite), an organic phase (~90% type I collagen), and water [2]. Proteins are assembled together to form a nanostructured extracellular matrix (ECM). The ECM acts as a prime cue that controls cell adhesion, proliferation, and differentiation of osteoblasts, osteocytes, and osteoclasts [3]. In this regard, development of a three-dimensional (3D), biodegradable scaffold having the aforementioned characteristics, which provides a specific environment and architecture for bone growth, is desirable in supporting bone tissue formation in vivo [4].

Bone tissue engineering scaffolds should be developed in accordance with various governing factors related to composition, structural features, and biological activity [5]. The basic structural framework could be ceramics, polymers, copolymers, or a combination; with non-toxic, biocompatible, bio-resorbable, and biodegradable properties, and smart, bioactive, and non-immunogenic characteristics [6]. At the same time, the scaffold should be biomimetic and bioinspired, with tailored architecture, customized shape with high porosity and pore interconnection, suitable surface topography, and good mechanical properties. Scaffolds with such properties for bone tissue engineering could be fabricated from suitable base materials by various methods [7,8,9]. Apart from material characteristics, various stem cells, including umbilical cord blood-derived stem cells, embryonic stem cells, muscle-derived stem cells, bone marrow-derived stem cells (BMSCs), and adipose tissue-derived stem cells, have received wide attention in bone tissue engineering, due to their diverse biological capability to differentiate into the osteogenic lineage. Among them, BMSCs and adipose tissue-derived stem cells are the most commonly used cell sources in bone tissue engineering [10].

In recent years, significant advancements in the development of novel biomaterials in terms of mechanical properties, osteo-conductivity, and osteogenic features have been achieved [11,12]. In this respect, the development of mechanically-stable scaffolds with good load-bearing ability is one of the greatest challenges, since inadequate mechanical support during bone regeneration could lead to poor osteo-integration [13]. Ceramic-based nanomaterials and other natural or synthetic polymeric-based materials have been used as bone regeneration scaffolds due to the ECM structural similarity in a bone-mimicking environment. Nanohydroxyapatite is considered one of the most widely explored ceramic nanomaterials for bone tissue engineering due to its strength, porosity, and most importantly, its resemblance to the native bone inorganic component. Many methods of nHAP preparation were reported in the literature, including biomimetic deposition, wet chemical deposition, electrodeposition, and sol-gel processes [14]. The physico-chemical properties of nHAP were shown to be dependent on the production methods and processing conditions [15]. However, a potential limitation of hydroxyapatite alone as a bone graft substitute is its low solubility, slow in vivo resorption profile, and brittle nature [16]. On the other hand, biocompatible, and biodegradable polymeric materials such as poly(lactic-*co*-glycolic acid) (PLGA) are inadequate to replicate the bone mechanical strength in the ideal porous form required for a scaffold, although they are much stronger in bulk form. Scaffolds integrated with PLGA microparticles or PLGA nanoparticles have been used for bone tissue engineering [17,18]. Nonetheless, they are inferior at mimicking the natural ECM of bone, compared to the highly biocompatible, but mechanically weaker, natural polymers, such as collagen, gelatin, alginate etc. [19,20,21]. It is not difficult to mimic the complex structural composition in hard tissues such as bone using those biologically-active natural polymers (i.e., collagen, gelatin), but the mechanical stability would be compromised. At the same time, relatively stronger ceramic or synthetic polymer-based scaffolds would provide a poor ECM mimicking environment, leading to slower bone regeneration.

Considering all the factors mentioned above, we focus on a systematic combination of a mechanically stable poly(lactic-*co*-glycolic acid) (PLGA)-based microsphere cavity with biologically active gelatin cryogel to form a hybrid scaffold having simultaneous load-bearing/bone regeneration potential. Both PLGA and gelatin was incorporated with a high percentage of nHAP, in order to induce osteo-inductive and osteo-conductive properties. A PLGA-nHAP microsphere cavity fabricated through heat sintering could generate high strength, while gelatin-nHAP cryogel can accelerate bone regeneration through the osteogenic nature of nHAP. Gelatin is readily assimilated by the body when used in vivo, and a combination of both nHAP and gelatin is expected to show increased osteo-conductivity and biodegradation, with relatively higher mechanical strength [22]. At the same time, nHAP-incorporated PLGA microspheres are not only osteo-conductive/inductive, but also stable constructs that can withstand load-bearing in bone, to a certain extent. PLGA-based microsphere scaffolds are reported to be good substitute materials in bone tissue engineering when used as a composite material with nHAP or tricalcium phosphate [23]. In short, we intend to develop a hybrid bone tissue engineering scaffold with both load-bearing and bone regeneration capabilities using a high-strength PLGA-nHAP microsphere cavity fitted with biomimetic gelatin-nHAP cryogel. The bone regeneration ability will be demonstrated in vitro and in vivo using BMSCs to repair tibia defects in rabbits.

## 2. Materials and Methods

### 2.1. Materials

PLGA with a lactide to glycolide ratio 85:15 was purchased from Green Chemical Inc. (Taipei, Taiwan). Calcium carbonate (CaCO_3_), sodium hydroxide (NaOH), poly(vinyl alcohol) (PVA), cetylpyridinium chloride, gelatin (type A from porcine skin, 300 bloom), and 2-morpholinoethane sulfonic acid (MES) were obtained from Sigma Aldrich (St Louis, MO, USA). Calcium hydrogen phosphate (Ca_2_HPO_4_·2H_2_O) was purchased from Showa (Tokyo, Japan). Dichloromethane (DCM, CH_2_Cl_2_) was procured from Alfa Aesar (Ward Hill, MA, USA), while fetal bovine serum (FBS) and Dulbecco’s Modified Eagle’s medium (DMEM) were purchased from Gibco (Waltham, MA, USA) and Invitrogen (Carlsbad, CA, USA), respectively. 1-Ethyl-3-(3-dimethylaminopropyl) carbodiimide (EDC) was procured from Acros Organics, Thermal Fisher Scientific (Geel, Belgium). 6-Diamidino-2-phenylindole dihydrochloride (DAPI) for nucleus staining was purchased from Life Technologies, Thermal Fisher Scientific (Carlsbad, CA, USA). All chemicals were used as purchased.

### 2.2. Preparation of Nanohydroxyapatite (nHAP)

Ca_2_HPO_4_·2H_2_O and CaCO_3_ were used to prepare nHAP particles through a chemical precipitation method reported in our earlier study [24]. CaHPO_4_·2H_2_O (0.86 g) and CaCO_3_ (0.335 g) were gently mixed in 2.5 M NaOH solution in a water bath maintained at 75 °C, and further reacted for 1 h. The reaction was completed through termination of hydrolysis by keeping the mixture in an ice bath. The resultant solution was centrifuged, washed multiple times with double distilled (DD) water, and dried at 70 °C for 24 h to obtain nHAP.

### 2.3. Preparation of PLGA-nHAP Microspheres

**Figure 1:**
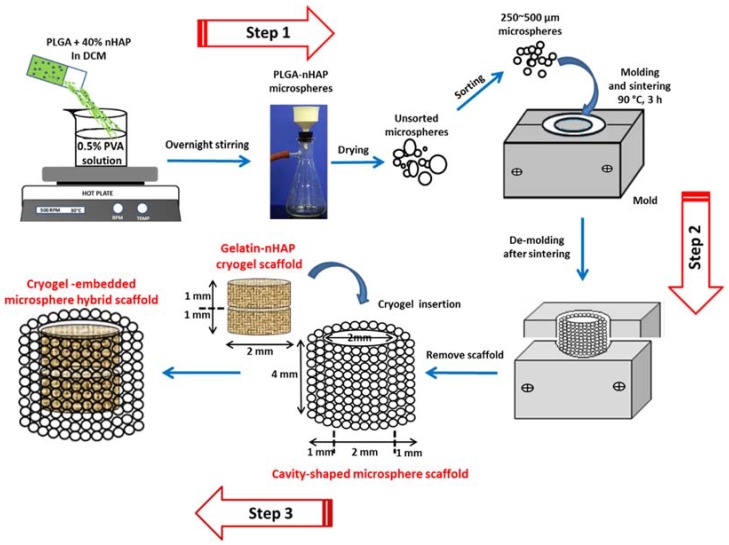
Preparation of PLGA-nHAP microspheres through emulsification using 0.5% PVA solution (**Step 1**) and further sorting and sintering of 250~500 μm PLGA-nHAP microspheres to obtain cavity-shaped microsphere scaffold with 2-mm inner diameter, 2-mm bottom thickness and 1-mm wall thickness (**Step 2**). Two gelatin-nHAP disk-shaped cryogel scaffolds (2 mm diameter × 1 mm thickness) were kept one over the other, and placed tightly inside the cavity of the microsphere scaffold to obtain a cryogel-embedded microsphere hybrid scaffold (**Step 3**).

Composite PLGA-nHAP microspheres were prepared by an emulsion method developed previously by the authors [25]. PLGA (2.1 g) was completely dissolved in 10 mL DCM. A quantity of 1.4 g of nHAP powder was slowly mixed with the PLGA solution under vortex to get a polymer mixture containing 40% (*w*/*w*) nHAP. The vortexed PLGA-nHAP solution was poured into the 1600 mL aqueous solution containing 0.5% (*w*/*v*) PVA as the surfactant, and stirred at 600 rpm. The mixing process was continued overnight to allow for microsphere formation, followed by washing in DD water, drying under vacuum, and storing in a dry cabinet (Step 1, Figure 1).

### 2.4. Fabricatin of Cavity-Shaped Microsphere Scaffolds

The unsorted PLGA-nHAP multi-sized composite microspheres obtained above were sieved using a stainless steel mesh, and selected microspheres (size 250~500 μm) were separately filled into a pre-fabricated, cavity-shaped, split-screwed stainless steel mold. The mold had a cup-shaped cavity with 4 mm height × 4 mm diameter, where a solid 2 mm height × 2 mm diameter metallic cylinder could be placed in the center of the cavity to obtain a negative-mold space of 2 mm height × 1 mm wall thickness. Composite PLGA-nHAP microspheres were layered first at the bottom of the cavity to 2 mm height, then the metallic cylindrical block was placed over it to maintain the bottom thickness of the to-be prepared cavity at 2 mm. More composite microspheres were placed in the 1 mm wall gaps of the cavity to the top of the mold, and the mold was screw tightened with a stainless steel top plate and sintered in an oven at 90 °C for 180 min. The obtained cavity-shaped microsphere scaffold was 4-mm height, 2-mm bottom thickness and 1-mm wall thickness, so that the central cavity would be 2 mm height × 2 mm diameter (Step 2, Figure 1). For optimization of the sintering conditions and to achieve the highest scaffold strength, cylindrical microsphere scaffolds (4 mm height × 4 mm diameter) were prepared at different sintering temperatures (80, 85 and 90 °C) and sintering duration times (60, 120 and 180 min), and subject to compression testing using a 250 N load cell with a cross-head speed of 0.02 mm/s in a Bose ElectroForce^®^ 5200 BioDynamic™ Testing Machine (Eden Prairie, MN, USA).

### 2.5. Characterization of nHAP, PLGA-nHAP Microspheres and PLGA-nHAP Microsphere Scaffolds

The morphology of nHAP in bulk form was observed by scanning electron microscopy (SEM) (S-3000N from Hitachi, Tokyo, Japan), whereas the characteristic Ca/P stoichiometric ratio was measured through elemental analysis by energy dispersive X-ray spectroscopy (EDS) (EX-250 from Horiba, Kyoto, Japan). The chemical structure of nHAP was observed by Fourier transform infrared spectroscopy (FTIR, Spectrum RX1 from Perkin-Elmer, Waltham, MA, USA) with a wavelength range of 4000 to 400 cm^−1^. Characteristic crystalline planes of nHAP were identified through a D5005 X-ray diffractometer (XRD, Siemens AG, Munich, Germany) fitted with a Cu-Kα source (λ = 1.541 Å) with a scanning speed of 2°/min from 10° to 60°. The size, morphology and surface characteristics of PLGA-nHAP composite microsphere scaffolds were observed through SEM, whereas the effective incorporation of nHAP in PLGA was evaluated through FTIR and XRD of the composite microsphere, with respect to nHAP and PLGA alone. In addition, the distribution of nHAP in PLGA microspheres was qualitatively evaluated through elemental mapping using SEM/EDS. Porosity of the scaffolds was determined through the ethanol displacement method [26]. The thermal stability of the samples was evaluated through thermogravimetric analysis (TGA) and differential thermal gravimetric (DTG) analysis by heating at 10 °C/min from 35 to 700 °C, using TGA 2050 (TA instruments, New Castle, DE, USA) under an inert atmosphere. The effect of nHAP in PLGA-nHAP microspheres was evaluated by plotting remaining weight percentage and derivative weight with respect to temperature (°C).

### 2.6. Preparation of Gelatin-nHAP Cryogel Scaffolds

A gelatin solution (8%) was prepared by dissolving pre-weighed gelatin flakes in an MES buffer (pH = 6.5) at 70 °C. Pre-weighed nHAP nanoparticles were dispersed in the gelatin solution and vortexed to get a gelatin-nHAP suspension with 2% nHAP (solution A). EDC was dissolved in a 10 mL MES buffer (pH = 6.5) to reach an initial concentration of 0.02 M (solution B). Solution A and B were mixed at an equal volume ratio to get a 4% gelatin/1% nHAP suspension in a bottom-capped 3 mL syringe (8.5 mm inner diameter) that served as the mold, and further mixed using a home-made vibration-free overhead spindle stirrer. The solution was slowly stirred to avoid air bubble formation, and the open end was closed with Parafilm Wrap. The syringe mold was immersed in a 95% ethanol bath kept at −16 °C for 16 h to complete the cryogelation process. The syringe mold was taken out of the bath after completion of the reaction, and the cryogel scaffold was allowed to recede through the bottom cap. The cryogel was cut into cylindrically shaped discs of 1 mm thickness, from which gel disks of 2 mm diameter × 1 mm thickness were made using a tissue puncher. All cryogel scaffolds were transferred to a −80 °C freezer for 24 h and further lyophilized to obtain macroporous gelatin-nHAP cryogel scaffolds containing 20% (*w*/*w*) nHAP.

### 2.7. Characterization of Gelatin-nHAP Cryogel Scaffolds

The prepared cryogel scaffold was characterized for its morphology using a field emission scanning electron microscope (FESEM, Hitachi, SU8010, Tokyo, Japan), whereas the elemental composition was estimated through EDS (Bruker AXS-5030, Billerica, MA, USA). Chemical compositions of nHAP-containing cryogels were identified through FTIR (Spectrum RX1, Perkin-Elmer, Waltham, MA, USA) and the crystalline planes were recorded using XRD (D5005, Siemens AG, Munich, Germany). Similar to microsphere scaffolds, porosity of the scaffolds was measured through the alcohol gradient method, while TGA/DTG (TGA 2050, TA instruments, New Castle, DE, USA) was performed from 35 to 700 °C, at a heating rate of 10 °C/min.

### 2.8. Fabrication and Mechanical Properties of the Cryogel-Embedded Microsphere Hybrid Scaffold

The sintered cavity-shaped microsphere scaffolds having respective 4 mm × 2 mm × 1 mm (height × bottom × wall thickness) dimensions were placed on a flat surface. Two pre-fabricated gelatin-nHAP cryogel scaffolds with 2 mm diameter × 1 mm thickness were inserted one over the other inside the cavity of the microsphere scaffold to obtain a cryogel-embedded microsphere hybrid scaffold (Step 3, Figure 1). The spongy nature of cryogels allowed tight embedding in the much harder and stronger microsphere cavity chamber. The mechanical properties of the cryogel scaffold and hybrid scaffold were determined from the stress (σ) vs. strain (ε) curves using a Bose ElectroForce^®^ 5200 BioDynamic™ Testing Machine (Eden Prairie, MN, USA).

### 2.9. In Vitro Studies

#### 2.9.1. Isolation and Culture of rBMSCs

Rabbit bone marrow-derived mesenchymal stem cells (rBMSCs) for cell culture studies were isolated as per the standard procedures reported earlier [27]. Young New Zealand white rabbits were anesthetized and 20 mL of blood was withdrawn from the bone marrow using an aspiration needle containing 5 mL heparin (anticoagulant). The procedures were approved by the Institutional Animal Care and Use Committee of Chang Gung University (IACUC Approval No. CGU15-022). The blood was mixed with phosphate buffer saline (PBS), centrifuged at 4 °C, and the supernatant was removed. The filtrate containing cell suspension was further mixed with an equal amount of cell expansion medium (20% FBS, 80% DMEM, 1% penicillin-streptomycin and 2 μg/mL fibroblast growth factor-2). Centrifugation was repeated and the supernatant was removed. The dark-red solution containing rBMSCs in the bottom layer was added to a T-75 flask containing 10 mL of cell culture medium and kept in a CO_2_ incubator maintained at 37 °C. Non-adherent cells were removed by changing the cell expansion medium every 5 days. The cells were further sub-cultured, and rBMSCs at passages 2 or 3 were used for all studies.

#### 2.9.2. Cell Proliferation and Alkaline Phosphatase (ALP) Activity

Cryogel-embedded hybrid microsphere scaffolds were sterilized in 75% ethanol followed by UV sterilization for 24 h. All scaffolds were rinsed with PBS three times, and kept in Nunc 24-well culture plates (Thermo Fisher Scientific, Carlsbad, CA, USA) for cell seeding. Samples were pre-wet with cell culture medium (90% DMEM, 10% FBS and 1% antibiotic/antimycotic) prior to cell seeding. An aliquot of 10 μL cell suspension containing 1 × 10^5^ rBMSCs was seeded on the cryogel scaffolds placed in the middle of the hybrid scaffold and maintained at 37 °C in a CO_2_ incubator for 4 h to allow cell adhesion. All the samples were transferred to another 24-well culture plate to remove unattached cells. Gelatin-nHAP cryogel scaffolds alone with the same dimensions as those used in the hybrid scaffold were seeded with the same cell number for comparison. The same cell culture medium (normal medium with no osteogenic factors) was used throughout the experiments, with medium change every two days. The DNA content was measured by DNA assays using Hoechst 33258 [28]. The intracellular alkaline phosphatase (ALP) activity of rBMSCs was measured using an ALP kit (SensoLyte^®^ pNPP ALP assay kit, AnaSpec, Freemont, CA, USA). Scaffolds were retrieved at the same evaluation time points as DNA content analysis, washed three times with PBS, and immersed in a cell lysis solution containing 500 μL of 0.1% Triton X-100 and 5 mM MgCl_2_. Samples were centrifuged at 13,000 rpm for 10 min at 4 °C, and 50 μL of the supernatant solution was mixed with an equal volume of 5 mM p-nitrophenyl phosphate solution prepared in 150 mM 2-amino-2-methyl-1-propanol buffer at room temperature for 30 min. All experiments were done in the dark, and the reaction was stopped by adding 50 μL of 0.2 N NaOH. The absorbance were measured at 405 nm using an ELISA reader, and the specific ALP activity per cell basis was determined by normalizing the ALP activity with DNA content, and expressed as ALP/DNA (ng/μg).

#### 2.9.3. Cell Mineralization

The scaffolds were taken out of the medium at 14 and 28 days, washed with PBS, and fixed using 4% glutaraldehyde and maintained at room temperature for 2 h. Samples were further washed with PBS three times and post-fixed with 1% osmium tetroxide (OsO_4_ in 0.1 M phosphate buffer) at room temperature for 2 h. PBS washing was applied another three times for a total of 20 min, followed by dehydration using alcohol gradient (50%, 70%, 80%, 90%, 95% and 100% alcohol). A critical point dryer was used to attain complete drying. Samples were mounted on carbon tape pasted aluminum holders and sputter-coated with gold at 30 mA for 60 s. The morphology of cells and mineralization on samples were monitored through SEM. The possibility of cell migration from the cryogel part of the hybrid scaffold to the surroundings was also assessed by SEM. Estimation on the mineral deposition by rBMSCs was performed through EDS analysis of the atomic percentage of elements in deposited minerals. The calcium content was verified through calcium ion (Ca^+^) quantification using Alizarin red S (ARS). Scaffolds were washed with PBS, fixed using a 2.5% glutaraldehyde solution for 2 h and submerged in an ARS solution (1 g ARS in 50 mL deionized water) for 1 h at room temperature. After the incubation period, samples were washed with DD water to remove excess dye, and treated with 1 mL of 10% cetylpyridinium chloride solution for 1 h to chelate calcium ions. The absorbance of the solution was read at 540 nm in an ELISA reader (Synergy HTX, BioTek, Winooski, VT, USA) normalized with DNA content, and reported as OD_540_/μg DNA [25].

#### 2.9.4. Immunofluorescent Staining of COL I and OCN

After cell culture, samples were rinsed with PBS and fixed with 4% formaldehyde in PBS for 30 min, followed by washing with PBS/0.1% Tween 20 (PBST) 3 times at 15 min each. One milliliter of HyBlock 1-min Blocking Buffer^®^ (Goal Bio, Taipei, Taiwan) was used to block nonspecific labeling and washed again with PBST. Samples were placed on a staining rack and separately incubated in either type I collagen (COL I) primary antibody (1:100 in PBST, mouse monoclonal anti-collagen I, Novus Biologicals, Littleton, CO, USA) or osteocalcin (OCN) primary antibody (1:100 in PBST, mouse monoclonal anti-osteocalcin, Abcam, Cambridge, UK) for 1 h. The treated samples were further rinsed with PBST for 20 min and separately incubated in FITC-conjugated goat anti-mouse IgG secondary antibodies (Jacksons Laboratories, Bar Harbor, ME, USA) for 1 h. Samples were then washed with PBST and stained with DAPI (50 μg/mL) for nucleus. All samples were quickly rinsed with PBST and imaged under a Leica TCS SP2 laser scanning microscope (Wetzlar, Germany) at an excitation/emission wavelength of 490/519 nm for FITC and 358/461 nm for DAPI. The blue-stained nucleus was used for cell counting, while the green-stained proteins were subject to quantification. The semi-quantitative estimation of COL I and OCN was done through PAX-it!^TM^ image analysis software (PAX-it, Villa Park, IL, USA) by analyzing the area percentage of the green fluorescence signal corresponding to COL I or OCN in each image.

#### 2.9.5. Quantitative Real-Time Polymerase Chain Reaction (qPCR)

The expression of osteogenic differentiation marker genes for COL I, OCN, and osteopontin (OPN) was examined using standard protocols of RNA isolation and cDNA synthesis [29]. RNA was isolated using TRIzol, and the corresponding solution was transferred to a 1.5 mL micro-centrifuge tube. Two hundred milliliters of chloroform was added to the cell suspension and vortexed for 15 to 30 s. The tube was further kept in an ice bath for 5 min and centrifuged at 11,000 rpm for 15 min. RNA was isolated, reacted with isopropanol in 1:1 ratio at −80 °C for 30 min, and the supernatant was removed again for further centrifugation at 11,000 rpm (4 °C) for 15 min. At 4 °C, 1 mL of 75% ice cold ethanol was added and mixed for 10 min and centrifuged at 11,000 rpm; this process was performed twice. The final supernatant solution was dried, and RNA was dissolved in 30 mL of diethyl pyrocarbonate-treated water at 55 °C for 15 min and reverse-transcripted into cDNA using SuperScript III RNase H. The quantitative real-time PCR was performed using a SYBR^®^ Green RT-PCR kit (SYBR Green I supermix) with a MiniOption detection system (CFD-3120, Bio-Rad, Hercules, CA, USA). Glyceraldehyde-3-phosphate dehydrogenase (GAPDH) was taken as the housekeeping control, and the results were analyzed using the GeneXpression Macro Chromo4 software program (Bio-Rad, Hercules, CA, USA) using the comparative threshold cycle method. Observed results were quantified using the 2^−ΔΔCt^ relative quantification method, and reported as relative mRNA expression after normalizing to the value on day 0.

#### 2.9.6. Biomechanical Testing

The biomechanical properties of the cell/scaffold constructs after in vitro culture were evaluated through compression testing using an ElectroForce^®^ 5200 BioDynamic™ testing machine (Bose, Eden Prairie, MN, USA). The samples were allowed to undergo a static compression testing at room temperature using a 250 N load cell with a cross-head speed of 0.02 mm/s. From the stress (σ) vs. strain (ε) curves, Young’s modulus (MPa) was calculated and plotted over time.

### 2.10. In Vivo Animal Study

Animal studies were performed as per the standards of the Association for Assessment and Accreditation of Laboratory Animal Care, and approved by the Institutional Animal Care and Use Committee of Chang Gung University (IACUC Approval No. CGU106-021). Male New Zealand white rabbits weighing 3 to 4 kg were selected for the study and kept in a single room in comfortable conditions for 10 days. Food and water were made available to all animals during the pre- and post-operative periods. All the animals were fed comfortably in their cages throughout the period till sacrificing. All the surgeries were performed under aseptic conditions in the laboratory. An intramuscular injection of Atropin (0.3 mg/kg) was given, followed by a general anesthesia using a mixture of Zoletil 50 (18 mg/kg) and Rompun 20 (1 mg/kg). Both tibias of the rabbit were shaved, and the cutaneous surface was disinfected with iodine solution prior to surgery. A longitudinal skin incision of 3 cm was made over both the tibias, a periosteal flap was elevated, and the medial surface of proximal metaphysis of the tibia was exposed. Four-millimeter-depth bone defects were created by drilling mid-diaphyseal, using a 4 mm diameter drill. Defects in tibias were treated with both cellular and acellular hybrid scaffolds of 4 mm × 4 mm (diameter × height) dimensions. A hybrid scaffold seeded with 1 × 10^5^ cells rBMSCs and cultured in cell culture medium for 14 days was used to fill the defect in the right tibia while the defect in the left tibia was filled with an acellular hybrid scaffold as a control. Both defect sites were completely occupied by the samples, and surgical incisions were closed in layers using 4-0 Ethicon sutures. The wounds were sterilized and dressed with gentamicin ointment to prevent infection, followed by antibiotic (gentamicin) intramuscular injection (3 mg/kg) for 3 days post-operation. The animals were returned to their cages and allowed to bear full weight, with no limitation of range of motion.

At 12 weeks post-operation, rabbits were euthanized by a lethal injection of pentobarbital (0.5 g per kg body weight). The tibias were subject to micro-computed tomography (μCT) examination using a SkyScan 1076/1174 μCT scanner (Bruker, Kartuizersweg, Belgium), and the implants were dissected out for gross evaluation. Both cellular and acellular implants were fixed in 10% formaldehyde, dehydrated, and embedded in paraffin, as per the standard protocols to make 10-μm slice sections on glass slides. Samples were then stained with hematoxylin and eosin (H&E), Masson’s trichrome, and immunohistochemical (IHC) staining of COL I and OCN. New bone formation from rBMSCs-seeded hybrid scaffolds in comparison with the acellular hybrid scaffolds was assessed by recording the images under an inverted optical microscope (Olympus IX-71, Tokyo, Japan).

### 2.11. Statistical Analysis

All quantitative data were expressed as mean ± standard deviation. Statistical analysis was performed using the one-way ANOVA LSD test to determine significant differences. A *p* value less than 0.05 was considered as statistically significant.

## 3. Results and Discussion

### 3.1. Characterization of nHAP

**Figure 2:**
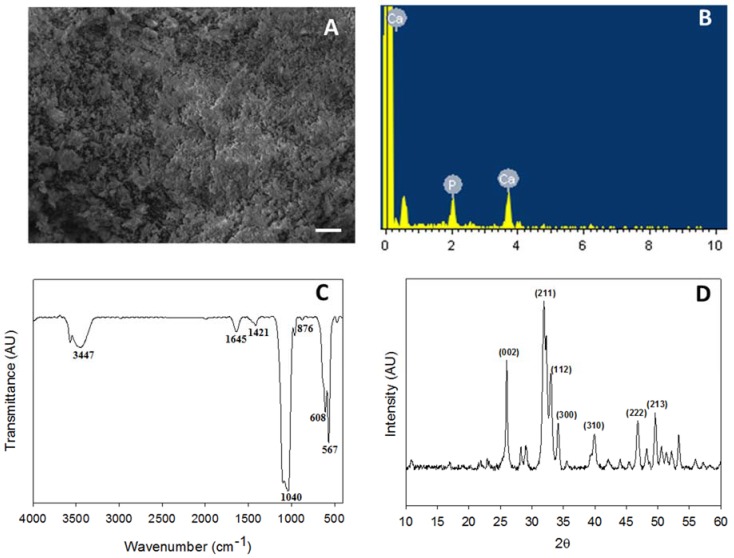
Characterization of nHAP by SEM (bar = 20 μm) (**A**), EDS (**B**), FTIR (**C**) and XRD (**D**).

The bulk morphology of nHAP was evaluated through SEM and a homogenous dispersion of nanoparticles was observed (Figure 2A). Particles had less agglomeration, with average particle size less than 50 nm, as per our previous observations [24]. EDS was performed to identify the elemental composition of the prepared nHAP, where atomic percentage of calcium (Ca) was 61.39 while phosphorous (P) was 38.61 (Figure 2B). The stoichiometric ratio of calcium to phosphate was 1.59 ± 0.17, which is close to the ideal value of 1.67 found in hydroxyapatite (Ca_10_(PO_4_)_6_(OH)_2_) in bones [30]. The characteristic stretching and bending vibrations associated with various functional groups in nHAP was confirmed from FTIR. The carbonate ion substitution was identified at the stretching band at 876 cm^−1^, whereas the phosphate stretching vibrations were observed at 567, 608, and 1040 cm^−1^. The band at 1421 cm^−1^ belonged to the hydroxyl group, while the respective sharp and broad bands at 1645 and 3447 cm^−1^ were due to the adsorbed moisture [31] (Figure 2C). The crystalline structure of nHAP was further evaluated through XRD analysis. The two prominent 2θ peaks observed at 26.0° and 32.0° correspond to the 002 and 211 plane, whereas the other minor peaks at 33.1° (112 plane), 34.2° (300 plane), 39.9° (310 plane), 46.8° (222 plane) and 49.5° (213 plane) further confirmed the formation of the apatite crystalline structure (Figure 2D). All peaks observed were compared to the JCPDC file number 090432 [24]. The crystallinity degree was calculated to be 0.81 for nHAP synthesized here [15].

### 3.2. PLGA-nHAP Composite Microspheres

**Figure 3:**
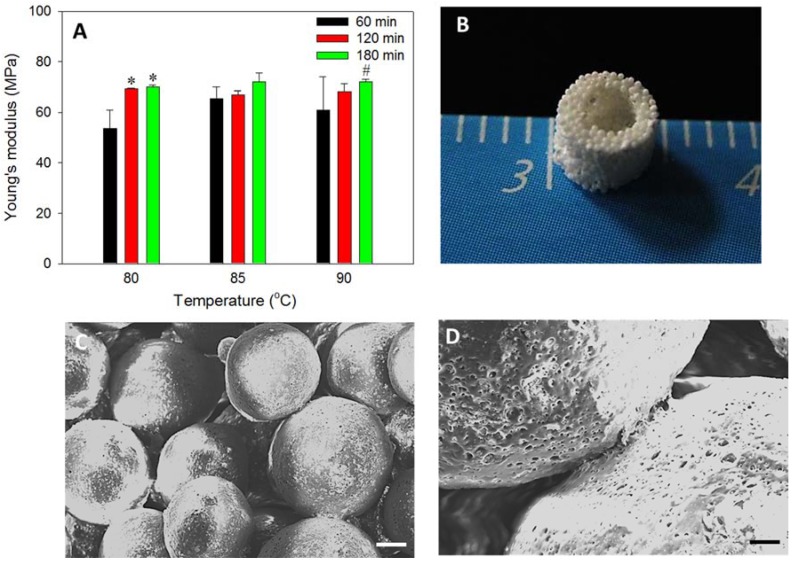
(**A**) Mechanical strength of the cavity-shaped microsphere scaffolds evaluated through the compression testing. * *p* < 0.05 compared to 60 min (80 °C), ^#^ *p* < 0.05 compared to 80 °C (180 min). (**B**) Gross view of the cavity-shaped PLGA-nHAP microsphere scaffold. (**C**,**D**) SEM images showed surface fusing of PLGA-nHAP microspheres in the cavity-shaped microsphere scaffold (**C**, bar = 100 μm; **D**, bar = 20 μm).

A modified method of PLGA microsphere preparation was used to fabricate composite microspheres at 40% nHAP content. In our earlier work, PLGA microspheres with 15% nHAP loading was successfully prepared, followed by sintering the PLGA-nHAP microspheres to bone tissue engineering scaffold for BMSCs osteogenic differentiation [25]. However, an attempt was made here to further enhance the osteogenic and osteo-conductive properties of PLGA-nHAP microspheres by increasing the nHAP loading in PLGA to a higher value (40%) during the emulsification process of microsphere preparation. The surfactant concentration (0.5% PVA) and mixing speed was maintained as before, and only the nHAP concentration was increased to enhance the ceramic nanoparticle concentration in PLGA-nHAP microspheres. However, the selection of sintering temperature and sintering time duration were considered prime parameters in sintered microsphere scaffold preparation, and an array of temperature-time studies was performed to identify the optimum temperature and time required for best surface fusing and mechanical integrity. Our earlier report used 85 °C for 90 min to obtain scaffolds with optimal properties [25]. However, in the current study, it was observed that the higher nHAP content (40%) in PLGA microspheres may lead to lower heat transfer rate, and thus, could deteriorate surface fusing; therefore, the identification of optimal parameters (temperature and time) is of real importance. The Young’s modulus of the sintered cavity-shaped scaffold prepared at various sintering temperature-time scales is shown in Figure 3A. Considering 85 °C as the reference point observed in earlier reports, a lower temperature (80 °C) and a higher one (90 °C) were tested for 60, 120, and 180 min sintering time. No significant difference in Young’s modulus was observed among any scaffolds, but a trend of higher strength at 180 min sintering duration time was observed, irrespective of temperature. Since the percentage of nHAP in PLGA microspheres is higher in the current system, a 90 °C sintering temperature for a duration of 180 min was selected for PLGA-nHAP microsphere scaffold fabrication.

### 3.3. Cavity-Shaped PLGA-nHAP Composite Microsphere Scaffold

The sorted microspheres (250–500 μm) were filled in a stainless steel mold, sintered at 90 °C for 180 min as shown in Step 2, Figure 1, and de-molded to obtain a porous and sturdy cavity-shaped scaffold, as shown (Figure 3B). The wall thickness and bottom capping was precisely maintained at 1 and 2 mm, respectively, while the inside cavity of 2 mm × 2 mm was impeccably generated without sharp edges. The uniform distribution of macro-pores in the cavity-shaped composite microsphere scaffold is clearly evident from the SEM image (Figure 3C), where surface sintering of microspheres led to pore formation. The fusing of PLGA microspheres at sphere intersection is more visible from the higher magnification SEM image (Figure 3D), with rough and irregular dots representing the presence of nHAP in the microsphere, as observed in our previous study [25].

**Figure 4:**
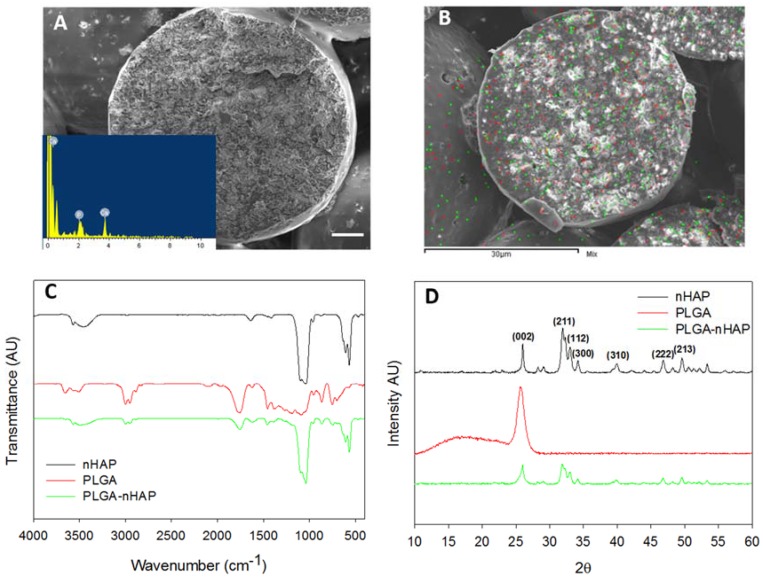
Characterization of cavity-shaped PLGA-nHAP microsphere scaffolds through SEM observation of scaffold cross section (**A**, bar = 50 μm), elemental mapping of Ca (green dots) and P (red dots) atoms (**B**), FTIR (**C**) and XRD (**D**).

The morphology of sintered microspheres is shown through cross-sectional SEM (Figure 4A). The size of the individual sphere is ~360 μm, with rough and irregular inner morphology, while the presence of nHAP in it was further proven through EDS (Figure 4A insert) with a Ca/P stoichiometric ratio of 1.61 ± 0.13, which is comparable with the nHAP value (1.59 ± 0.17) shown in Figure 2B [32]. The elemental mapping was used to verify the distribution of Ca and P atoms of nHAP in the microsphere (Figure 4B). With green dots representing Ca and red dots denoting P, both atoms were found to uniformly distribute in the interior, as well as on the surface of the microsphere. All results thus confirmed the uniform encapsulation of nHAP in the PLGA-nHAP microsphere, which is essential to trigger osteo-conduction during bone regeneration.

From FTIR analysis, the cavity-shaped microsphere scaffolds were spectrally characterized in comparison with nHAP and PLGA (Figure 4C). The stretching band of nHAP at 876 cm^−1^ was due to carbonate ion substitution, whereas the phosphate stretching vibrations were observed at 567, 608, and 1040 cm^−1^. The hydroxyl peaks of nHAP were visible at 1421 and 3573 cm^−1^. Considering the spectrum of PLGA, characteristic peaks were shown for the alkyl group in the broad range of 2948–2994 cm^−1^, in addition to the hydroxyl vibrations at 695 and 3642 cm^−1^ and C=O stretching at 1752 cm^−1^. Asymmetric and symmetric stretching bands of C–C were also observed in the wavenumber range of 1097–1163 cm^−1^ [33]. The PLGA-nHAP microsphere scaffold had all the characteristic peaks due to both nHAP and PLGA, as evidenced from the characteristic phosphate stretching vibrations of nHAP at 567, 608, and 1043 cm^−1^. The hydroxyl peaks of nHAP at 1421 and 3573 cm^−1^ also matched those in the spectrum of the PLGA-nHAP scaffold. The alkyl group stretching observed for PLGA at 2948–2994 cm^−1^ was also found in the microsphere scaffold. The asymmetric and symmetric C–C stretching of PLGA merged with phosphate stretching vibrations of nHAP at 1040 cm^−1^ to become a broad peak at 975–1173 cm^−1^, confirming the presence of nHAP in PLGA-nHAP microspheres.

The crystalline structure of the microsphere scaffold was further evaluated through XRD analysis in comparison with nHAP and PLGA (Figure 4D). nHAP displayed prominent 2θ peaks at 26.0° (002) and 32.0° (211), along with the other minor peaks at 33.1° (112 plane), 34.2° (300 plane), 39.9° (310 plane), 46.8° (222 plane) and 49.5° (213 plane) while PLGA had only one characteristic peak at 2θ = 25.7° [25]. Therefore, the PLGA-nHAP microsphere scaffold showed all diffraction peaks corresponding to both nHAP and PLGA.

When considering the tissue engineering applicability of a scaffold, porosity is the most relevant factor. Tissue engineering commonly encompasses the use of 3D scaffolds to provide a suitable microenvironment for incorporation of cells or growth factors to regenerate damaged tissues or organs. Materials having high porosity could enable the effective release of genes, proteins, or cells, and provide good substrate characteristics for waste and nutrient exchange [34]. However, the mechanical property that is important in maintaining the structural stability of the biomaterial is often compromised as the result of increased porosity [35]. In this approach, a highly macroporous, microsphere-based cavity scaffold is introduced to support the cryogel core of the hybrid scaffold so that it can function as an efficient mechanical supporter for the relatively weaker cryogel core part that is endowed with osteogenic and osteo-conductive properties for bone regeneration. The porosity of the cavity-shaped microsphere scaffold was measured to be 47% ± 0.9%, which is adequate enough to allow nutrient transfer, and at the same time, maintain the mechanical integrity for load bearing.

**Figure 5:**
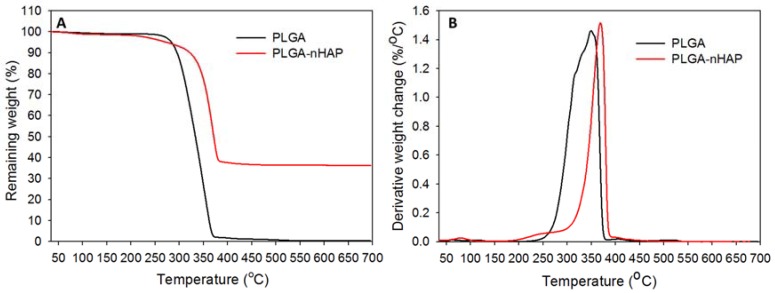
(**A**) Thermogravimetric analysis (TGA) and (**B**) differential thermogravimetric (DTG) analysis of PLGA microspheres and PLGA-nHAP microsphere scaffold.

The thermal stability of the microsphere scaffold was verified through TGA (Figure 5A) and DTG (Figure 5B), in contrast with virgin PLGA microspheres. Both scaffolds had stable decomposition resistance up to ~256–262 °C, with no significant weight loss. However, a steep decomposition pattern was observed for both samples above 300 °C, resulting in 43% remaining weight for both samples at 340 °C. Virgin PLGA microspheres could retain only 2.98% of its initial weight after 370 °C, and completely decomposed at 500 °C. Most of the biodegradable polymeric materials undergo fast decomposition at higher temperatures through charring, as observed for virgin PLGA microspheres [24]. However, for the composite microsphere scaffold, the sudden decomposition was halted at 348 °C, and maintained a plateau pattern throughout the test till 700 °C, to attain a final remaining weight of 36.4%. The TGA results thus emphasize not only the thermal stability of nHAP containing composite microsphere scaffold, but also justified the higher actual loading percentage of nHAP (36.4%) in PLGA-nHAP microspheres, in comparison with a theoretical value of 40%. The shift in peak decomposition values from ~350 °C of virgin PLGA microspheres to ~370 °C of the microsphere scaffold indicated that embedding nHAP in PLGA could enhance the thermal stability of the composite microsphere, and correlated with the early decomposition nature of virgin PLGA microspheres (Figure 5B). In short, nHAP incorporation in PLGA microspheres increased the thermal stability of the cavity-shaped PLGA-nHAP composite microsphere scaffold with high nHAP loading close to the theoretical nHAP loading value.

### 3.4. Gelatin-nHAP Cryogel Scaffold

Composite gelatin-nHAP cryogel scaffolds were prepared by the process of cryogelation at −16 °C. The compositions of gelatin-nHAP cryogels were designed based on the optimal cryogel properties that could fulfill the minimal criteria of scaffolds intended for bone tissue engineering. Osteo-inductive scaffolds have been fabricated by incorporating HAP into scaffolds [36,37]. HAP was also believed to be osteo-conductive [38]. Osteo-conductivity and osteo-inductivity are two important properties for bone grafts. When nHAP was entrapped in crosslinked gelatin matrix in gelatin-nHAP cryogel scaffolds, the nHAP concentration was sufficient enough to accelerate osteo-induction towards bone formation. Apart from osteo-induction, incorporation of ceramic particles can enhance the mechanical properties of the scaffold, which is very much needed for scaffolds with load bearing applications such as bone substitutes [39]. Though there is a possibility of weakening of the scaffold due to the higher nHAP content and relatively lower porosity compared to gelatin cryogel without nHAP, use of cavity-shaped microsphere scaffold, which is intended to use as a mechanical supporting matrix for cryogel, could meet the challenge. Generally, cryogels with low gelatin content are soft and elastic but mechanically inadequate, while gelatin concentrations greater than 5% offer an unsatisfactorily high cross-linking rate and brittle nature [26]. Considering these factors, use of 4% gelatin for cryogel fabrication was attempted here to fabricate an ideal cryogel scaffold.

**Figure 6:**
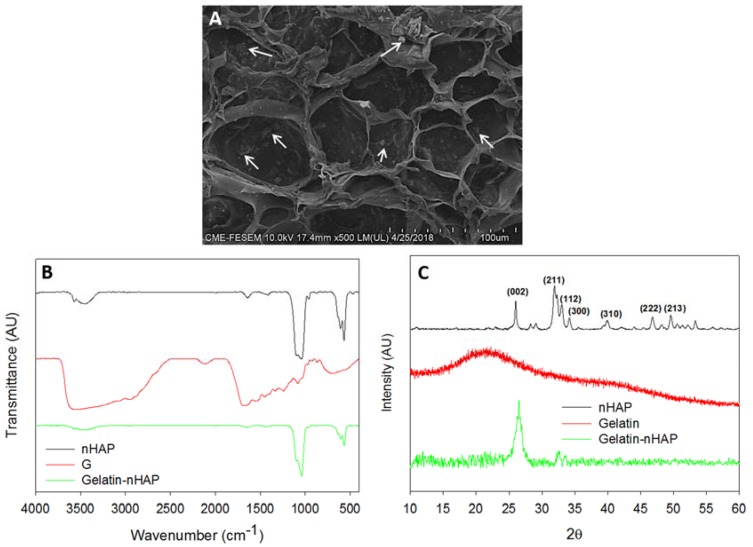
Characterization of gelatin-nHAP cryogel scaffold through SEM (**A**), FTIR (**B**) and XRD (**C**). The white arrows indicate nHAP embedded in gelatin.

The morphology of prepared composite cryogel scaffold was examined by SEM and shown in Figure 6A, where the scaffold had an open, interconnected macroporous morphology, with an average pore size of 65 ± 29 μm. The macroporous morphology was sufficient for cell migration into the interior, while the inter-connective pores could channel the waste and nutrient transfer similar to native environment. The embedded nHAP inside the cryogel scaffold was uniformly distributed throughout the scaffolds, as denoted by the white arrows in Figure 6A. The presence of nHAP in the composite cryogel was further confirmed from EDS and the atomic percentages of Ca and P were 6.88 and 4.52, respectively, from which the Ca/P ratio was calculated to be 1.52 and close to the theoretical value.

The chemical structure of gelatin-nHAP cryogel was further examined by FTIR, through which the functional groups present in nHAP and gelatin in gelatin-nHAP cryogel scaffold could be identified (Figure 6B). For nHAP, the characteristic stretching and bending vibrations were observed at 876 cm^−1^, representing the carbonate (CO_3_^2−^) substitution; whereas the phosphate (PO_4_^3−^) stretching vibrations were observed at 567, 608 and 1040 cm^−1^. The hydroxyl peaks of nHAP were visible at 1,421 and 3573 cm^−1^. The FTIR spectrum of gelatin showed that the broad peak from 3611 to 3147 cm^−1^ was due to N–H stretching of secondary amide, while the band at 1683 cm^−1^ was due to C=O stretching. Peaks at 1447 and 1531 cm^−1^ were from N–H bending, 686 cm^−1^ due to the N–H out of plane wagging, and minor peaks at 1021 and 2924 cm^−1^ originated from the C–H stretching [40]. In composite gelatin-nHAP cryogels, all characteristic peaks corresponding to gelatin and nHAP appeared, indicating the successful incorporation of nHAP into the composite cryogel. The broad peak in the composite cryogel from 3634 to 3250 cm^−1^ was due to the overlapping bands of the hydroxyl group of nHAP and the N–H stretching of gelatin. The peak at 1645 cm^−1^ corresponded to the C=O stretching of gelatin while 1421 cm^−1^ originated from the hydroxyl peak of nHAP. The characteristic phosphate stretching vibrations of nHAP at 567, 608 and 1040 cm^−1^ were also observed in the cryogel scaffold.

Crystalline peaks of nHAP in gelatin-nHAP cryogel scaffolds were identified through XRD (Figure 6C), using gelatin and nHAP as reference samples for comparison. Gelatin displayed a typical XRD pattern of the partially crystalline structure with a peak at 2θ = 21.76°, originating from the triple-helical crystalline structure in collagen and gelatin [41]. nHAP showed an apatite crystalline structure from the reflection peaks observed from 2θ values at 26.0° (002) and 32.0° (211), along with the other minor peaks at 33.1° (112), 34.2° (300), 39.9° (310), 46.8° (222), and 49.5° (213). When nHAP was incorporated into gelatin, all crystalline peaks of nHAP still existed, but the broad gelatin peak at 21.76° was not evident. The reduction of gelatin peak intensity was due to the screening effect of strong nHAP peaks in the composite cryogels, as mentioned in earlier literature [42]. It is noted that nHAP peaks in the cryogel also slightly broadened and weakened compared with original nHAP, implying low crystallinity for the cryogel scaffold. In short, the XRD study validated the formation of composite cryogel scaffolds through nHAP incorporation. Porosity of the cryogel was measured through the alcohol gradient method as described for composite microsphere scaffolds. Porosity is one of the crucial factors in the designing of tissue engineering scaffold for cell seeding, where cellular infiltration leads to the regeneration process. The porosity of the cryogel scaffold was measured to be 82.1% ± 2.6%. The much more porous nature of the cryogel scaffold compared to the microsphere scaffold was beneficial for rapid cell distribution during cell seeding, with further cell proliferation and differentiation of rBMSCs into osteoblasts [26].

**Figure 7:**
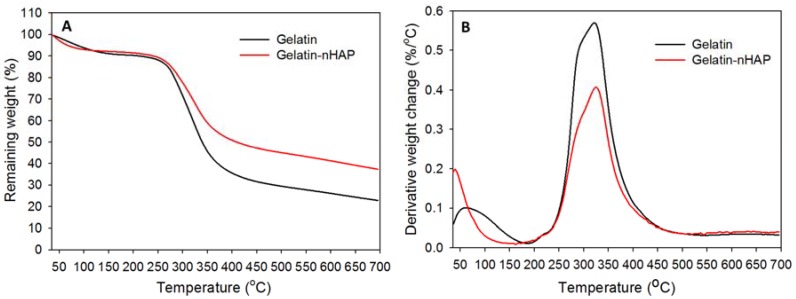
(**A**) Thermogravimetric analysis (TGA) and (**B**) differential thermogravimetric (DTG) analysis of gelatin and gelatin-nHAP cryogels.

Thermal stability was estimated from TGA (Figure 7A) and DTG thermograms (Figure 7B) by heating from 35 to 700 °C. Both pure gelatin and gelatin-nHAP cryogels showed comparable initial decomposition from 220 to 258 °C (Figure 7A) and a peak thermal decomposition temperature of ~323 °C (Figure 7B) [39]. However, the increased thermal stability induced by the composite cryogel was clearly visible from 87.7% (gelatin) and 89.6% (gelatin-nHAP) remaining weight within the initial decomposition period and 59.5% (gelatin) and 68.6% (gelatin-nHAP) remaining weight at the peak temperature. Continued thermal decomposition initiated at ~315 °C for gelatin and ~332 °C for gelatin-nHAP cryogel and continued to get a final residual weight of 22.4% for gelatin and 37.3% for gelatin-nHAP at 700 °C. The higher residual weight of the nHAP-containing gelatin cryogel scaffold could be attributed to the ceramic nanoparticle (nHAP) in it, which did not disintegrate at 700 °C. From the weight difference at 700 °C, the nHAP content in gelatin-nHAP cryogel scaffold could be estimated to be ~15% (*w*/*w*), which is close to the theoretical value (20%). The lower nHAP content could be due to loss of the nanoparticles during the washing step when preparing the composite gelatin-nHAP cryogel scaffold.

### 3.5. Cryogel-Embedded Hybrid Scaffold

**Figure 8:**
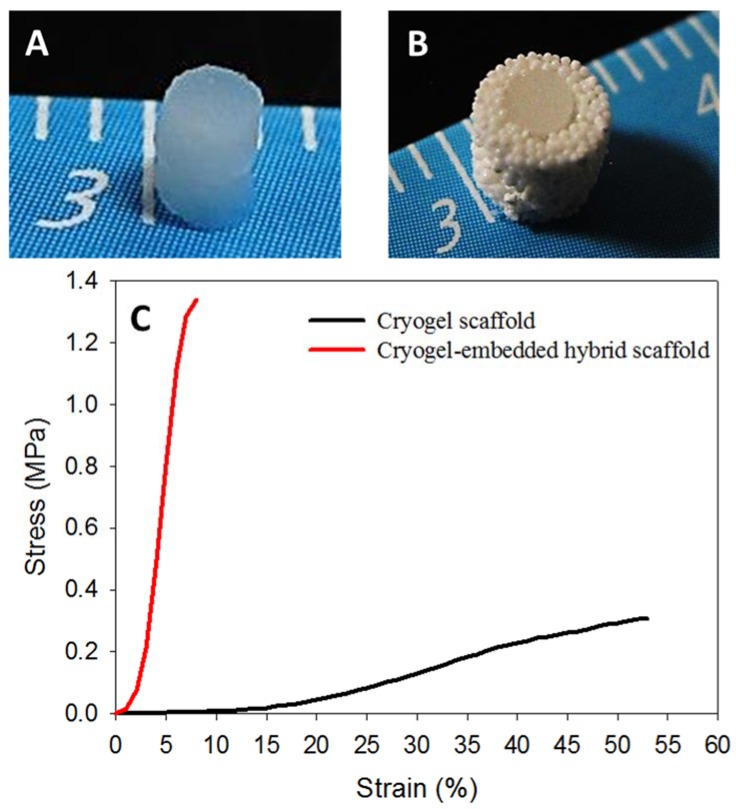
Gross view of the gelatin-nHAP cryogel scaffold (**A**) and the cryogel-embedded microsphere hybrid scaffold (**B**). (**C**) The stress-strain curves of the gelatin-nHAP cryogel scaffold and the cryogel-embedded microsphere hybrid scaffold.

Considering the weak mechanical properties of cryogel scaffolds, a new approach of scaffold matrix re-enforcement through high strength microsphere cavity design could drastically enhance the bio-mechanical output of a tissue engineering scaffold. Presence of nHAP in both microsphere and cryogel scaffolds can accelerate bone regeneration through its osteo-inductive and osteo-conductive natures. In bone tissue engineering, cryogel-based scaffolds are too week to meet the load-bearing requirement while fulfilling bone regeneration needs [43]. At the same time, surface curvature of sintered microspheres causes a relatively slower cell proliferation rate than cryogel, but is ideal in terms of load-bearing. Considering all these factors, two composite disk-shaped cryogel scaffolds with 2-mm diameter and 1-mm thickness (Figure 8A) were kept, one over the other, inside the cavity of the composite microsphere scaffold (Step 3, Figure 1), to develop a cryogel-embedded microsphere hybrid scaffold with ideal load-bearing properties for bone regeneration (Figure 8B). The spongy nature of cryogel allowed its tight embedding inside the high strength PLGA-nHAP microsphere cavity, enabling the idyllic design for transportation of waste and nutrient through pores of sintered microspheres, while accelerated bone regeneration could be supported from the rBMSCs-seeded gelatin-nHAP cryogel scaffolds in the core. To confirm the approach used here to enhance the strength of cryogel scaffolds, the mechanical properties of both scaffolds were characterized by compression testing; the stress-strain curves are shown in Figure 8C. As expected, the ultimate stress of the hybrid scaffold (7.75 ± 0.50 MPa) was 25 times that of the cryogel scaffold (0.31 ± 0.03 MPa), while the Young’s modulus of the hybrid scaffold (20.03 ± 2.01 MPa) was 21 times that of the cryogel scaffold (0.98 ± 0.19 MPa). The prepared hybrid scaffold was further compared with the cryogel scaffold for in vitro culture of rBMSCs.

### 3.6. In Vitro Studies

#### 3.6.1. Cell Proliferation and ALP Activity

**Figure 9:**
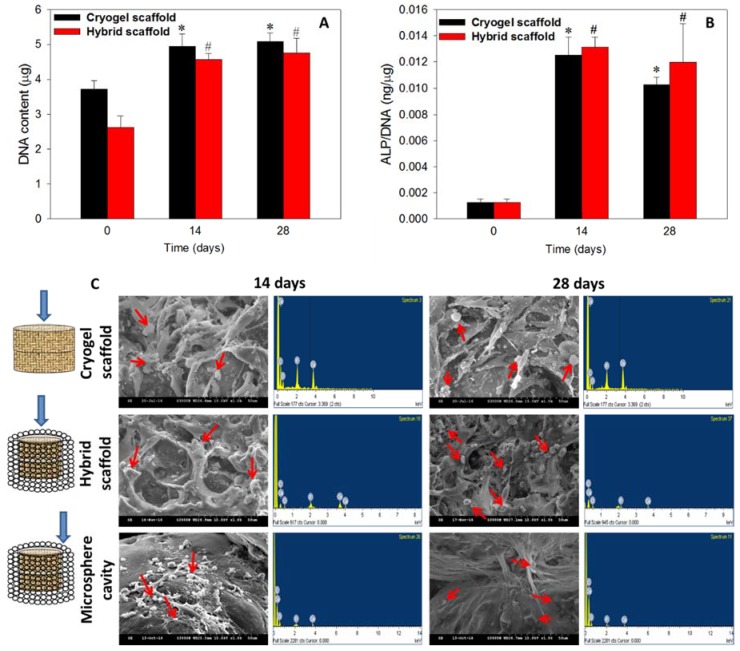
The DNA content (**A**) and ALP activity (**B**) of rBMSCs cultured in cryogel and hybrid scaffolds. (**C**) Qualitative assessment of mineralization through SEM and quantitative estimation of mineral composition by EDS of the cryogel scaffold, the cryogel part in the hybrid scaffold and the microsphere cavity in the hybrid scaffold. The blue arrows indicate places where SEM/EDS samples were taken. The red arrows indicate mineral deposition from cell differentiation. * *p* < 0.05 compared to cryogel scaffold on day 0, ^#^ *p* < 0.05 compared to hybrid scaffold on day 0.

The rationale behind the selection of the cryogel scaffold, in addition to the hybrid scaffold for in vitro assessments, was to determine if there is any significant change in cell response of the cryogel scaffold after embedding in the microsphere scaffold to form the hybrid scaffold. Stem cells are believed to have better proliferation and differentiation capabilities in a 3D micro-environment, owning to mimicking the natural architecture in bone and cartilage tissue [44,45]. The rBMSCs cultured in scaffolds were analyzed for cell proliferation through DNA content (Figure 9A) while osteogenic differentiation was assessed quantitatively through ALP activity assays (Figure 9B). Compared to day 0, both cryogel and hybrid scaffolds had significantly higher DNA contents (*p* < 0.05) on days 14 and 28, but no statistical difference was found between them (Figure 9A). Indeed, the cell number plateaued after day 14, as evidenced from the statistically non-significant increase of DNA content on day 28, due to the osteogenic differentiation of rBMSCs [46]. It could be concluded that as rBMSCs are induced into the osteoblast lineage in the presence of nHAP, cells will become more mature and exhibit growth arrest due to limited cell proliferation of differentiated stem cells.

Bone formation is a two-stage process, starting with cell proliferation, and followed by extracellular matrix (ECM) maturation and mineralization. A high-level expression of ALP by the cells will occur first during the differentiation stage of bone followed by ECM maturation, and the matured ECM will mineralize at the end. Hence, the elevation of ALP activity and production of mineralized matrix are two major specific events during osteogenic differentiation of BMSCs [47]. This can be supported from the normalized ALP activity on days 14 and 28, where a drastic increase of ALP activity (~13 folds) was observed when compared to day 0 (Figure 9B). The similar ALP production rates on days 14 and 28 were consistent with the early osteogenesis marker nature of ALP [48]. The saturation of cell proliferation on day 28 was directly related to the differentiation potential of both scaffolds containing nHAP [49], even in the absence of osteogenic induction factors in the cell culture medium. Demonstrating also an osteo-inductive property, nHAP was considered to be one of the best materials to trigger stem cell differentiation to bone [50], thereby justifying the higher ALP content at later stages of cell culture with a saturation of cell proliferation. The highest expression of ALP on day 14, and a trend of reaching saturation at later stages, justifies the differentiation pattern of rBMSCs in presence of nHAP, as reported earlier [51,52].

#### 3.6.2. Cell Morphology and Mineralization

SEM/EDS analysis was performed to evaluate the morphology of rBMSCs in the cryogel scaffold, the cryogel part in the hybrid scaffold, and the wall of the microsphere cavity in the hybrid scaffold. Cells in the first two scaffolds showed flat spread cell morphology on days 14 and 28, while cells migrated to the cavity-shaped microsphere scaffold had a mixed round-flat morphology with mineralized nodules throughout the matrix (Figure 9C). A high density of cell population was observed within the cryogels, confirming homogenous cell growth and differentiation which was ideal for regeneration. Relatively fewer cells appeared at the inner wall of the microsphere cavity, confirming the migration of cells from the cryogel core to the exterior through the macropores of the cryogel. However, the migrated cells spread well on the curved surface of microspheres, while those on the microsphere intersections lead to a bridge between two sphere surfaces to fill the pores in a manner that was ideal for tissue regeneration [25]. The rounded white spots denoted by red arrows are the mineral deposition from cell differentiation, while long, spindle-shaped cells represent the cellular filopodial bridging inside the scaffolds. Basically, the initiation of mineralization is associated with the expression of the ALP marker where nucleation starts with the deposition of Ca^2+^ ions, and thus, results in calcification in the local environment [53]. A downstream cell differentiation factor from the hydrolysis of phosphate esters occurs due to the elevation in the mineralization of the cell ECM. Therefore, the high level ALP expression on days 14 and 28 (Figure 9B) was consistent with the mineralization of rBMSCs shown in Figure 9C.

Though mineralization can be well assessed qualitatively through the observation of substrate surface-based deposition through SEM [45,54], a measurement on the presence of calcium and phosphate is mandatory to prove the mineralization in vitro. In this regard, elemental analysis by EDS would be an easy tool for the quantitative estimation. Cortical bone contains calcium phosphate in the form of hydroxyapatite with an ideal Ca/P ratio of 1.67, for which a similar ratio of mineral deposition is much preferable during bone regeneration. The EDS spectra of minerals deposited by rBMSCs were included in Figure 9C, from which the corresponding Ca/P atomic ratios were calculated and shown in Table 1. The relatively higher level of Ca/P ratio on day 28 could be due to the higher secretion rate of Ca^2+^ ions by rBMSCs, compared to day 14. Thus, cell morphology and mineralization confirmed the differentiation of rBMSCs toward bone, in the presence of osteo-inductive/conductive nHAP. Taken together, rBMSCs seeded in the cryogel part of the hybrid scaffold exhibited comparable cell proliferation and osteogenic differentiation potential as cells seeded in the cryogel scaffold. Hence, embedding gelatin-nHAP cryogels in a microsphere cavity was shown to retain the cellular response and bone regeneration capability of the scaffold while simultaneously meeting the need for bone repair in load-bearing situation. Thus, only the hybrid scaffold was chosen for further cell differentiation studies.

#### 3.6.3. Calcium Content

**Figure 10:**
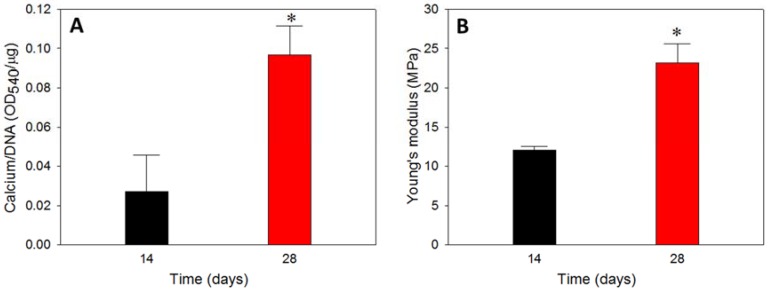
The calcium content (**A**) and Young’s modulus (**B**) of rBMSCs-seeded hybrid scaffolds cultured in vitro for 14 and 28 days. * *p* < 0.05 compared to day 14.

The calcium content was used to cross confirmed the calcium-based mineralization of rBMSCs, where rBMSCs in hybrid scaffolds exhibited a drastic increase in calcium content on day 28 compared to day 14 (Figure 10A). Typically, calcium deposition of stem cells starts in the later stages of cell proliferation, and therefore, the duration of culture period is crucial in determining the extent of calcium deposition [55]. The calcium estimation was done through a binding of calcium ions in mineralized ECM to form an ARS-calcium complex in a chelation process. The higher calcium content on day 28 implied that the time-dependent cell mineralization produced more Ca^2+^ binding sites for ARS, which can be justified from the higher Ca/P ratio for all scaffolds on day 28, shown in Table 1, as well as in the SEM images, shown in Figure 9C. The cell proliferation from DNA content and ALP production (Figure 9) could be also correlated with the calcium quantification, where the former had a plateau of cell growth after 14 days, implying cell differentiation through mineralization, while the latter displayed a higher level of mineralization inducing ALP marker. All results thus confirmed the differentiation of rBMSCs to osteoblasts through mineralization, where calcium deposition is the primary process.

#### 3.6.4. Biomechanical Properties

To determine the effect of mineral deposition and enhanced mineral-rich ECM production of rBMSCs after osteogenic differentiation, the Young’s modulus of cell/hybrid scaffold constructs after 14 and 28 days in vitro culture was determined by compression testing (Figure 10B). Considering the degradation characteristics of PLGA microspheres, an in vitro culture period of 28 days was not enough to initiate the hydrolysis of the sphere, which would lead to mechanical weakening of the scaffold [25]. Nonetheless, the cell/hybrid scaffold construct displayed a 1.93 times higher Young’s modulus on day 28 compared to day 14, implying that the enhanced stiffness occurred due to mineral deposition in the pores of the microsphere cavity from the migrated cells in the cryogel part. The macroporous nature of the cryogel scaffold mostly disappeared 28 days after in vitro culture (Figure 9C), implying using cryogel scaffold alone would lead to reduced mechanical properties at later culture periods. The cryogel scaffold was reported to display decreasing Young’s modulus with culture time [56]. Indeed, with the fast degradation rate expected for a cryogel scaffold formed from crosslinked gelatin, the cryogel scaffold is not expected to sustain high load-bearing in vivo as the deposited minerals would have a negligible effect in enhancing the strength. Therefore, the ideal requirement of a bone substitute is acquired through the successful embedding of weak strength/fast degradation cryogel scaffold inside the high strength/slow degradation microsphere cavity for cell seeding to develop a hybrid scaffold with excellent load-bearing capability.

#### 3.6.5. Gene Expression

**Figure 11:**
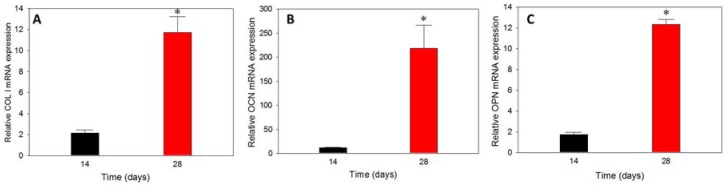
The relative (to day 0) mRNA expression of COL I (**A**), OCN (**B**) and OPN (**C**) of rBMSCs-seeded hybrid scaffolds cultured in vitro for 14 and 28 days by rea-time qPCR analysis. * *p* < 0.01 compared to day 14.

The substrate-dependent analysis on gene expression endorsed the effect of osteo-conductive or osteo-inductive materials in controlling the up-down gene regulation of various osteogenic marker proteins at early, mid, and later stages of cell differentiation [57]. The prominent osteogenic differentiation marker genes are ALP, COL I, OCN, and OPN and their presence could be verified from the relative mRNA expressions of rBMSCs cultured in hybrid scaffolds at different durations. ALP belongs to the early stage differentiation, COL I to the mid-stage, while OCN and OPN are expressed at the mid-later stage of stem cell differentiation. The relative (to day 0) mRNA expression of COL I, OCN and OPN is shown in Figure 11. COL I had relatively lower mRNA expression on day 14, but increased 5.5-fold on day 28, confirming the regeneration towards the osteogenic lineage. A similar trend was observed for OCN and OPN as expected, where the relative mRNA expression on day 28 was 18.7-fold and 7.2-fold that of day 14, respectively. The higher increase of mRNA expression for OCN and OPN coincided with their later gene expression natures than that of COL I. The presence of nHAP in microspheres, as well as in the cryogel part of the hybrid scaffold, have crucial roles in inducing the differentiation cycle, and further accelerate towards bone regeneration even in the absence of osteogenic factors in the medium. The gene expression results could be correlated with the early results of plateauing of cell proliferation and increasing of ALP activity, mineralization of rBMSCs after day 14, and higher level of deposited calcium.

#### 3.6.6. COL I and OCN Content by Immunofluorescent Staining

**Figure 12:**
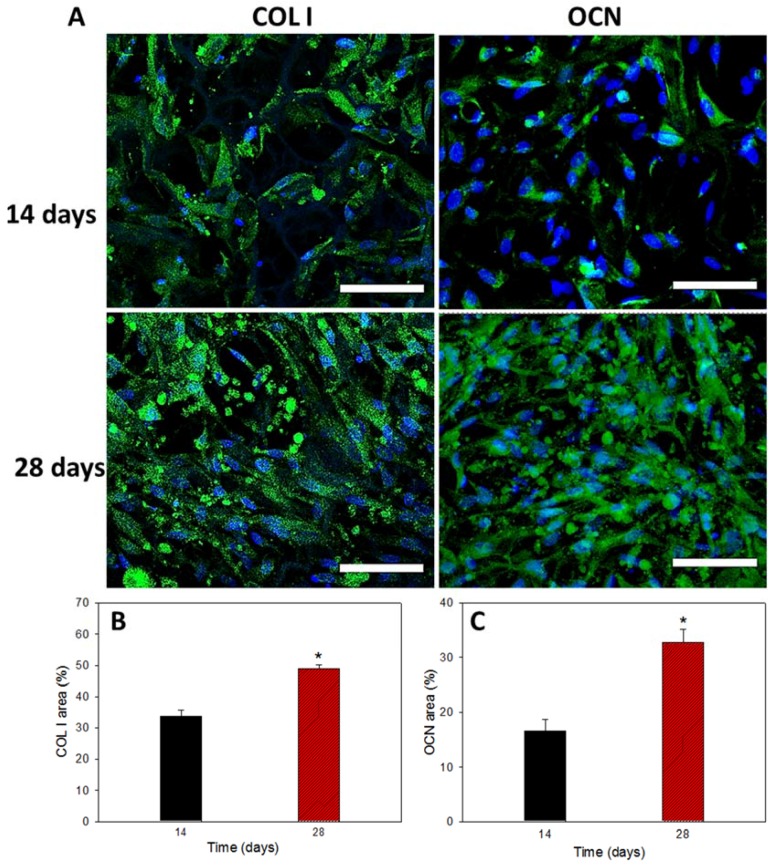
Immunofluorescent staining images using DAPI for cell nucleus and FITC-conjugated antibody for type I collagen (COL I) and osteocalcin (OCN) (**A**) (bar = 300 μm). The semi-quantitative analysis of COL I (**B**) and OCN (**C**) were obtained by analysis with PAX-it!^TM^ software. * *p* < 0.05 compared to day 14. Bar = 75 μm.

The mRNA expression of osteogenic marker genes observed from qPCR studies was further confirmed form marker protein production form immunofluorescent (IF) staining of COL I and OCN (Figure 12A). Both COL I and OCN are bone-specific proteins synthesized by osteoblasts, which represent good markers for osteogenic maturation of rBMSCs. Green fluorescence represents the proteins and blue is the DAPI-stained nucleus. On day 14, the samples showed partial distribution of COL I and OCN within the hybrid scaffold, but much denser fluorescent intensity was observed on day 28, indicating that more osteogenic protein (COL I and OCN) was produced from rBMSCs in the scaffold to validate stem cell differentiation into osteoblasts in vitro. The cells maintained spread morphology at both time points, as observed in mineralization, while similar nuclear-staining signals in blue confirmed cell growth arrest during cell differentiation (Figure 9A). A quantitative estimation of protein expression was performed through PAX-it!^TM^ image analysis software, which could precisely detect objects by shape, size, color or other criteria [58]. The area of the green color intensity above a chosen threshold value was selected and computed with reference to the total image area for semi-quantitative determination of COL I and OCN contents. As shown in Figure 12B,C, production of COL I or OCN was increased from 14 to 28 days. The area percentage of COL I on day 28 increased 1.43 folds compared to day 14. Similarly, the area percentage of OCN on day 28 increased 1.96 folds. The earlier COL I protein production in comparison with OCN is consistent with the mid-stage and mid-later stage expression of the respective bone marker genes (Figure 11).

### 3.7. In Vivo Animal Studies

**Figure 13:**
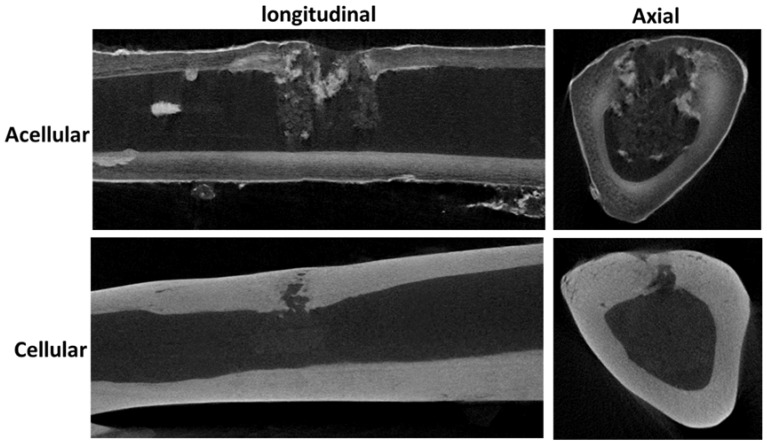
The micro-computed tomography (μCT) images of tibia defects in longitudinal and axial directions after repaired with acellular and cellular hybrid scaffolds 12-week post-operation.

Based on the in vitro results, acellular and cell-seeded hybrid scaffolds were implanted in the tibias of rabbits for bone regeneration study. The right tibia defect was implanted with the rBMSCs/hybrid scaffold as the cellular group, while the left tibia was used as a control to be repaired with an acellular hybrid scaffold. From 12-week post-operation μCT images, the implanted acellular hybrid resulted in minimum bone regeneration, as judged from the discontinuous bone mineral density at the implantation site, visible from both longitudinal and axial μCT images (Figure 13). However, a dramatic difference in bone regeneration was observed for the cellular group at the same time point. The bone defect site repaired with the rBMSCs/hybrid scaffold construct showed substantial increase in bone mineral density due to bone regeneration, shown from both longitudinal and axial views.

**Figure 14:**
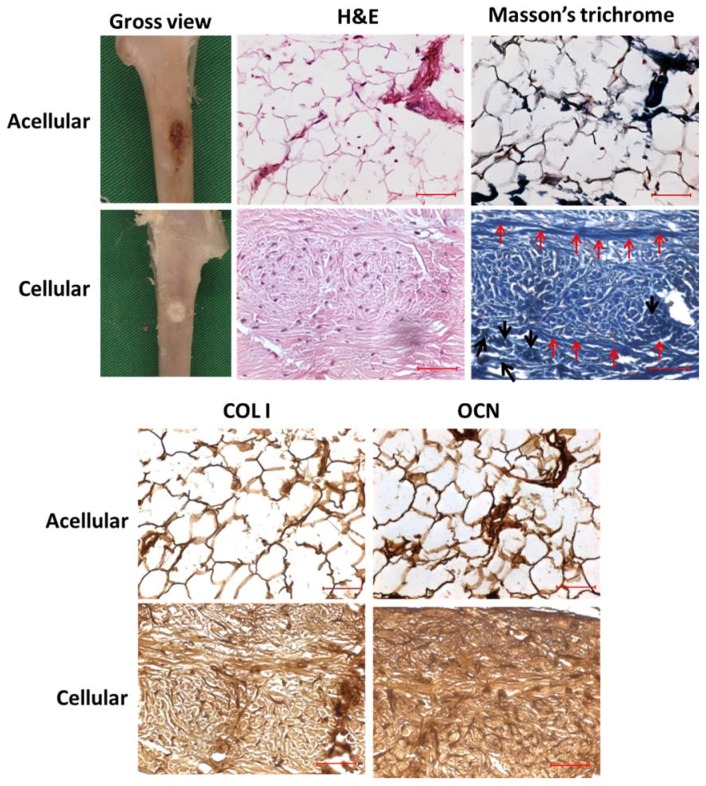
The gross view, H&E and Masson’s trichrome staining and immunohistochemical (IHC) staining of COL I and OCN of the tibia defects repaired with acellular and cellular hybrid scaffolds 12-week post-operation. The red arrows indicate the osteoid formation while the black arrows denote the existence of osteoblasts in Masson’s trichrome staining for cellular sample. Bar = 50 μm.

From gross observation of the retrieved tibias, the defect site implanted with the cellular sample had normal tissue appearance, while the defect site implanted with the acellular scaffold had a ruptured morphology, which might be due to incomplete and inadequate bone regeneration (Figure 14). Both defect surroundings had normal tissue appearance with no swelling or necrosis. The cellular sample appeared whiter in color compared to the acellular sample, showing the faster regeneration and distribution of bone tissue with it, while the dark part in the acellular sample demonstrated insufficient tissue regeneration due to the absence of rBMSCs.

The H&E staining endorsed drastic change in cell number as rBMSCs proliferated in the cellular sample in comparison with the acellular control sample (Figure 14). Uniform distribution of cells was found while ECM was oriented in a native fashion. As seen in the figure, the acellular scaffold displayed neither cell proliferation nor ECM production, and the empty scaffold cross-section morphology pointed to a lack of tissue regeneration. Few cell nuclei appeared in the section of acellular sample; this could be due to migrated osteoblasts or fibroblasts post-operatively from the surroundings. rBMSCs differentiation was further verified through Masson’s trichrome staining (Figure 14). The cellular scaffold showed deep blue staining throughout the section, while the acellular scaffold displayed only white background, due to the absence of cells, and thereby, insignificant regeneration in it. The similarity in stain intensity and morphology of both H&E and Masson’s trichrome staining further confirmed the different regeneration potential of the hybrid scaffold in the absence and presence of rBMSCs. In Masson’s trichrome staining, the deep blue-colored array of longitudinal stain in the sections of the cellular scaffold disclosed the osteoid formation (red arrows), while the cluster of cells denoted by black arrows endorsed the existence of osteoblasts [59,60]. When rBMSCs differentiate into osteoblasts, osteoids (organic portion of the bone matrix) will be embedded in the newly formed tissue matrix, and thus, could be proved through staining for connective tissues. The presence of limited blue-stained regions in the acellular scaffold might be due to cell infiltration from the surroundings, as was also observed in the H&E staining of the acellular sample.

In connection with H&E and Masson’s trichrome staining, the major components of osteoid, the fiber type COL I, and the ground substance OCN could be determined through immunohistochemistry to confirm the osteoblast phenotype. COL I and OCN are the respective mid and mid-later stage markers in osteogenic differentiation. In the IHC staining of COL I (Figure 14), the cellular sample displayed intense brown stains compared to the acellular scaffold, confirming the continuous proliferation and osteogenic differentiation of stem cells in vivo. Moreover, the homogenous distribution of COL I stain intensity in the cellular sample proved its significant dominance in cell differentiation toward bone through a uniform pathway, due to the interconnected macroporous morphology of the hybrid scaffold. That the brown stain intensity of OCN in cellular scaffold was much denser than COL I (Figure 14) could further support the late stage up-regulation of the OCN gene, compared to the early-mid stage expression of COL I. The relatively lesser staining on the acellular scaffolds for OCN was consistent with other staining results of the cellular scaffold, which justifies the lack of bone regeneration in the absence of stem cells. Histology and IHC staining confirm that the cell-seeded hybrid scaffold provided an effective tissue-engineered construct for bone replacement, revealing its simultaneous load-bearing and bone regeneration capability.

## 4. Conclusions

We prepared a novel hybrid bone tissue engineering scaffold with a cavity-shaped PLGA-nHAP microsphere scaffold embedded with gelatin-nHAP cryogels, which could be used for bone regeneration in load-bearing conditions. The chemical compositions of a nHAP, PLGA-nHAP microsphere scaffold, and a gelatin-nHAP scaffold were confirmed, and the physico-chemical properties were characterized in detail. That the hybrid scaffold showed 25-fold higher ultimate stress and 21-fold higher Young’s modulus than the cryogel scaffold indicated a high strength hybrid scaffold had been successfully developed. The in vitro cell culture studies endorsed cell growth arrest after day 14, with a concomitant rapid increase in ALP concentration due to osteogenic differentiation of rBMSCs in cryogel scaffolds and hybrid scaffolds. The successful osteogenesis in both scaffolds was also supported by mineralization of rBMSCs and SEM/EDX analysis. Calcium quantification and compression tests of the cell-seeded hybrid scaffold further proved its mechanical stability. Gene expression analysis revealed the upregulation of bone-specific marker genes COL I, OCN, and OPN, and thus, indisputably justified bone regeneration using the rBMSCs-seeded hybrid scaffold. This was also supported by the production of bone marker proteins COL I and OCN from IF staining. Through 12-week in vivo implantation experiments to repair load bearing tibia defects in rabbits, the μ-CT and histological analyses could cross-confirm the cell differentiation pattern and bone regeneration of rBMSCs in the hybrid scaffold in vivo. This study thus proves the efficacy of a microsphere-based cavity scaffold in aiding the load-bearing function of a bone tissue engineering scaffold, while the cryogel-based part could accelerate bone regeneration through the osteogenesis of rBMSCs in the presence of nHAP. Considering the dual function of the hybrid scaffold developed here without any compromise, it would be useful as a novel scaffold for bone tissue engineering.

## Figures and Tables

**Table 1:** The Ca to P atomic ratio of deposited minerals in the cryogel scaffold, the cryogel part in the hybrid scaffold and the microsphere cavity in the hybrid scaffold after in vitro culture for 14 and 28 days.

Scaffold	Day 14	Day 28
Cryogel	1.58 ± 0.05	1.72 ± 0.04 *
Hybrid	1.74 ± 0.05	1.91 ± 0.11 *
Microsphere	1.73 ± 0.06	2.02 ± 0.25 *

* *p* < 0.05 compared to day 14.
